# POSTER SESSION 3: Abstracts 666–947

**DOI:** 10.1002/pmf2.12027

**Published:** 2025-01-05

FRIDAY

January 31, 2025

10:30 AM – 12:30 PM

